# Mechanism of Improving Aspirin Resistance: Blood-Activating Herbs Combined With Aspirin in Treating Atherosclerotic Cardiovascular Diseases

**DOI:** 10.3389/fphar.2021.794417

**Published:** 2021-12-17

**Authors:** Yixi Zhao, Shengjie Yang, Min Wu

**Affiliations:** ^1^ Comprehensive Department, Guang’anmen Hospital, China Academy of Chinese Medical Sciences, Beijing, China; ^2^ Graduate School, Beijing University of Chinese Medicine, Beijing, China

**Keywords:** blood-activating, traditional Chinese medicine, aspirin resistance, atherosclerotic cardiovascular diseases, mechanism

## Abstract

Atherosclerotic thrombotic disease continues to maintain a high morbidity and mortality rate worldwide at present. Aspirin, which is reckoned as the cornerstone of primary and secondary prevention of atherosclerotic cardiovascular diseases (ASCVDs), has been applied in clinics extensively. However, cardiovascular events continue to occur even though people utilize aspirin appropriately. Therefore, the concept of aspirin resistance (AR) was put forward by scholars, which is of great significance for the prediction of the clinical outcome of diseases. The pathogenesis of AR may be incorporated with low patient compliance, insufficient dose, genetic polymorphism, increased platelet transformation, inflammation, and the degenerative changes and calcification of platelets. The improvement of AR in the treatment of ASCVDs has gradually become a research hot spot in recent years. Traditional Chinese medicine (TCM) regards individuals as a whole and treats them from a holistic view, which has been found to have advantages in clinical studies on the treatment of AR. Many kinds of blood-activating TCM have the effect of improving AR. The potential mechanism for the improvement of AR by blood-activating herbs combined with aspirin was explored. The combination of blood-activating herbs and aspirin to improve AR is likely to turn into a hot topic of research in the future.

## Introduction

Atherosclerotic cardiovascular diseases (ASCVDs) have a high incidence in the world and are the main cause of morbidity and mortality in China (The interpretation of, 2017). The increase in platelet reactivity, platelet activation, aggregation, and interaction with surface molecules is closely related to ischemic stroke, thrombosis, and cardiovascular diseases (CVDs) (Kannan et al., 2019; Khodadi, 2020; Wiśniewski et al., 2020). Aspirin, an acetylated salicylate, can irreversibly inhibit the conversion of arachidonic acid (AA) to thromboxane A2 (TXA2) by acetylating the serine 529 site of platelet cyclooxygenase-1 (COX-1), and then inhibit platelet production and play an antithrombotic role. Aspirin is widely used in clinics as the cornerstone of primary and secondary prevention of CVDs (Welsh et al., 2019; Nudy et al., 2020; Rocca and Patrono, 2020; Mogul et al., 2021). Nevertheless, a large number of studies have shown that about 2–4% of cardiovascular ischemic events, such as myocardial infarction, stroke, and death still reoccur every year after the rational use of drugs (Eikelboom et al., 2017; Sabatine et al., 2017; Schwartz et al., 2018). Patients with CVD who are at high risk of aspirin-induced bleeding were challenged by novel evidence of aspirin tolerance poses (Santos-Gallego and Badimon, 2021). The conception of aspirin resistance (AR) has been proposed, and the improvement of AR in the treatment of ASCVDs has gradually become a research hot spot in recent years (Locāne et al., 2019).

## Conception of AR and Current Research Studies

### Conception of AR

Aspirin is the representative medicine of antiplatelet aggregation (Ornelas et al., 2017). However, thrombosis events still occur after patients take aspirin in clinical practice, which is called AR (Paven et al., 2020). Henry et al. (2010) evaluated the 2- to 24-h peak and trough biological efficacy of daily low-dose aspirin in 150 patients with stable coronary heart disease (CHD). Light transmittance concentration (LTA) induced by 0.5 mg/ml AA was measured. It was found that AR appeared in one quarter of the patients. At present, AR is generally defined as the expected effect of antiplatelet aggregation which does not appear after patients regularly take conventional dose of aspirin, and laboratory indicators show that platelet activity or accumulation rate is not ideal, resulting in increased risk of cardiovascular events (Paseban et al., 2020). A study conducted on 126 Asian Indian patients with stable CHD found that 36% of patients had no response to aspirin (Chadha et al., 2016). In a systematic review and meta-analysis of 65 studies involving 10,729 patients, the overall prevalence of AR in CVD patients defined by the laboratory was 24.7% (95% CI 21.4–28.4). The risk of it was higher in women than in men, with a ratio of 1.16 (95% CI 0.87–1.54) (Ebrahimi et al., 2020). These pieces of evidence suggest that AR is common clinically and may affect the therapeutic efficacy.

### Detection and Significance of AR

There are no official diagnostic or regulatory guidelines for the AR concept (Ferreira et al., 2020). The determination of platelet function is a significant method to estimate the clinical prognosis of patients with AR. The *in vivo* platelet function test is prothrombin time. The *in vitro* platelet function test included the determination of the thromboxane and aspirin metabolite thromboxane B2 (TXB2) (Yassin et al., 2019). However, the AR standard most commonly used and accepted by researchers is that the average aggregation of 10 μmol/L adenosine diphosphate (ADP) is greater than 70%, and the average aggregation of 0.5 mg/ml AA is greater than 20% as proposed by Gum et al. (2001). Mohring et al. (2017) detected the formation of thromboxane induced by AA through enzyme-linked immunosorbent assay (ELISA) and the AA-induced antiplatelet effect of aspirin by the LTA method. The results showed that there was a non-linear correlation between the formation of thromboxane and the maximum value of AA-induced LTA aggregation (Spearman’s rho R = 0.7396; 95% CI 0.7208–0.7573, *p* < 0.0001). Receiver characteristics analysis and Youden’s J statistics showed that 209.8 ng/ml was the optimal cutoff value for thromboxane ELISA to detect high on-treatment platelet reactivity to aspirin (area under the curve: 0.92, *p* < 0.0001, sensitivity: 82.7%, specificity: 90.3%). This study showed that the thromboxane formation examined by ELISA is reliable for detecting high on-treatment platelet reactivity to aspirin.

The detection of AR has important implications to predict the clinical outcome of the disease. A 5-year follow-up study of 465 patients treated with aspirin found that multivariate logistic regression analysis showed a high association between AR and cardiovascular events (adjusted odds ratio, 4.28; 95% CI: 1.64 11.20; *p* = 0.03) (Chen and Chou, 2017). Wang et al. (2019) conducted a systematic review and meta-analysis of 35 clinical trials involving 19,025 patients with CHD to explore the relationship between the laboratory test AR and endpoint events. They found that the risk of all-cause death [7.9 vs. 2.5%, OR = 2.42 (95% CI 1.86–3.15, *p* < 0.00001)] and target vessel reconstruction [4.5 vs. 1.7%, OR = 2.20 (95% CI 1.19–4.08, *p* = 0.01)] in aspirin non-responders was higher than that in aspirin responders, indicating that AR has a good predictive effect on clinical outcomes. Li et al. conducted a systematic review of nine prospective studies including 1889 confirmed adherence patients with CHD who insisted on taking aspirin to explore the relationship between AR and the risk of major adverse cardiovascular events (MACEs). The results illustrated that the incidence of MACEs in patients with AR was significantly higher than that in patients with aspirin sensitivity (odds ratio 2.44, 95% CI 1.81 to 3.30; *p <* 0.000001). The risk of MACEs in the laboratory AR patients with CHD was 2.4 times higher than that in aspirin-sensitive patients (Li et al., 2014).

### Mechanism of AR Formation

**FIGURE 1:**
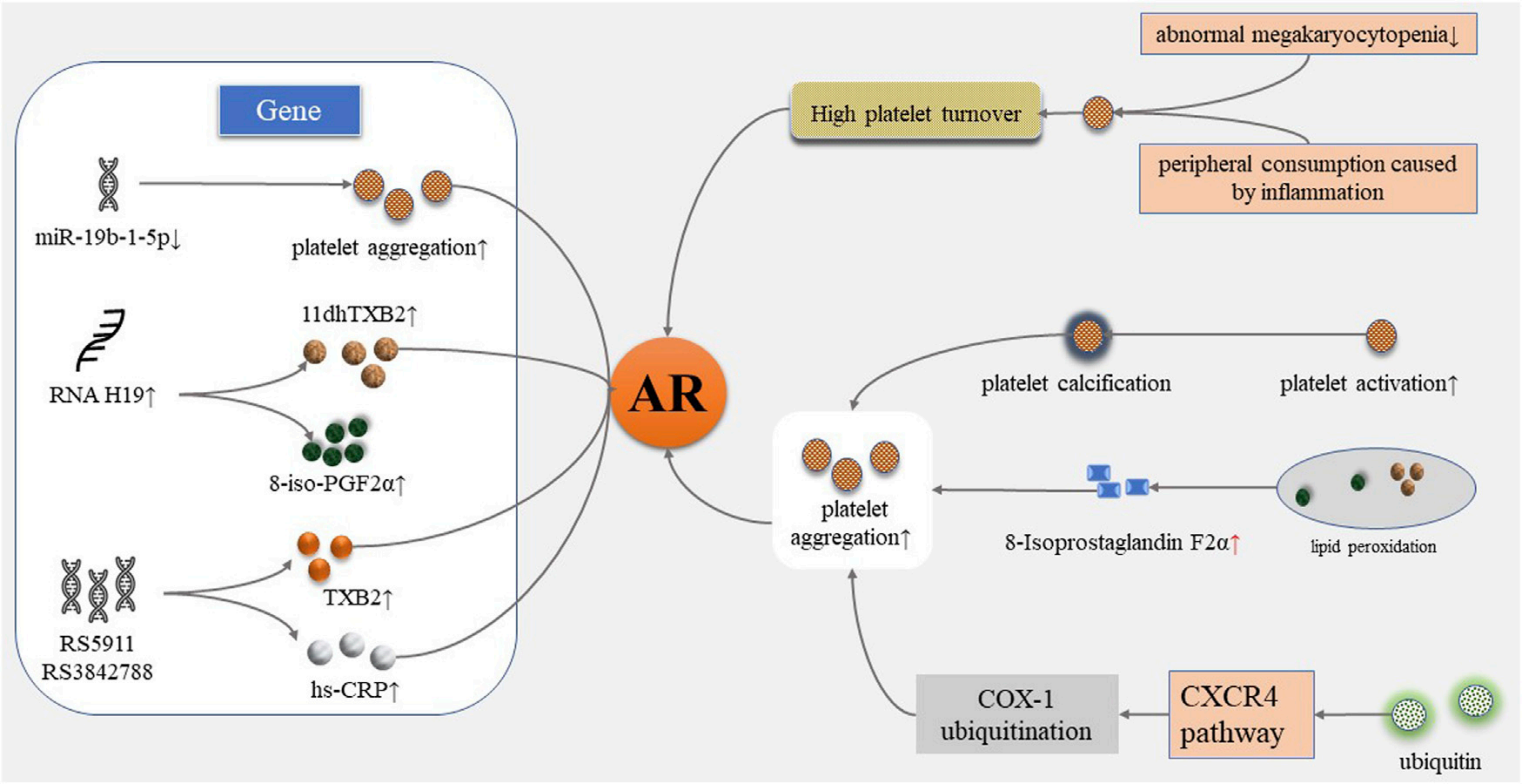
Mechanism of aspirin resistance. AR, aspirin resistance; 11dhTXB2, 11-dehydrogenation thromboxane B2; 8-iso-PGF2α, 8-iso-prostaglandin F2α; hs-CRP, high-sensitivity C-reactive protein; CXCR4, CXC chemokine receptor 4; COX-1, cyclooxygenase-1.

AR may be caused by many factors and involves multiple complex mechanisms. AR was found to be related to patients’ low compliance and insufficient drug dosage in clinics. Molecular studies have shown that the mechanisms of AR involves platelet gene polymorphism, increased platelet conversion rate, inflammation, and other mechanisms (Figure 1) (Floyd and Ferro, 2014; Du et al., 2016).

### Patients’ Incompliance to Aspirin

Grinstein and Cannon (2012) considered that low patient adherence to aspirin was the main reason for the failure of aspirin treatment and thus affected the analysis of AR. Dawson et al. (2011) used aspirin to investigate compliance in 90 ischemic stroke patients and 90 control subjects. Platelet function was assessed using standard definitions of resistance using platelet function analyzer (PFA)-100 and rapid platelet function analyzer (RPFA) devices. Urine levels of aspirin metabolites were measured by high-performance liquid chromatography to confirm treatment adherence. The results indicated that poor compliance accounted for nearly half of the cases of aspirin failure. The key point of AR research is to evaluate the compliance objectively. Cuisset et al. (2009) recruited 136 patients who underwent coronary stenting to explore the compliance of aspirin. The maximum intensity of AA-induced aggregation (AA-Ag) during hospitalization and a month after discharge was detected; aspirin non-responders were defined as AA-Ag > 30%.19 patients (14%) were identified as non-responsive 1 month after discharge, and AA-Ag in the population was significantly higher than that during hospitalization (15.3 ± 23 vs. 7.5 ± 10%, *p* = 0.0004). Only one person did not respond after receiving aspirin, suggesting that these changes were due to non-adherence. Schwartz, K. A. defines this class of patients as “inadequately responsive to aspirin” and believes that such patients are at increased risk of vascular events, and suggests that future studies should focus on improving compliance and reducing the risk of future cardiovascular events (Schwartz, 2011).

### Inadequate Dose of Aspirin

Doses of aspirin for primary and secondary prophylaxis of thrombosis in ASCVD vary from country to country and are usually aimed at achieving analgesic effects rather than achieving antithrombotic effects by complete acetylation of COX-1 (Linden and Tran, 2012).

Low-dose aspirin (75–150 mg) is a long-term effective antiplatelet aggregation therapy (Collaboration, 2002). Quinn, M. J. et al. compared the effects of low-dose (<150 mg) and medium-dose (≥150 mg) aspirin on the incidence of patients with unstable angina pectoris or acute myocardial infarction (AMI) within 6 months in the Global Use of Strategies to Open Occluded Coronary Arteries (GUSTO) IIb and Platelet Glycoprotein IIb/IIIa in Unstable Angina: Receptor Suppression Using Integrelin Therapy (PURSUIT) trials. It was found that high-dose aspirin was associated with a reduced incidence of MI [HR 0.79 (95% CI 0.64 to 0.98), *p* = 0.03] (Quinn et al., 2004). Lee, P.Y. et al. observed 468 patients with stable CHD who regularly took aspirin 80–325 mg for 4 weeks and found that the incidence of AR was higher with daily aspirin dose ≤100 mg than with daily aspirin dose of 150 and 300 mg (30.2 vs. 16.7% vs. 0%, *p* = 0.0062) (Lee et al., 2005). Vivas, D. et al. researched the effect of an additional dose of 100 mg aspirin on platelet function and the proportion of aspirin non-responders in 141 patients with CHD who took 100 mg aspirin on a long-term treatment using PFA-100. The incidence of aspirin non-response decreased from 50.7% (95% CI 42.4–59) to 35.0% (95% CI 27.3–43.2), illustrating that the additional dose of aspirin had a significant effect on aspirin non-response (Vivas et al., 2011). Perrier-Cornet, A. et al. observed that the AR rate decreased from 94 to 47% in patients with myeloid proliferative tumor after changing the aspirin dose from 75 mg once daily to twice daily (Perrier-Cornet et al., 2018).

The dosage of aspirin is still controversial. Haley, S.P. et al. reviewed 12 randomized controlled trials (RCTs) and found that aspirin was not dose-dependent on the incidence of bleeding or ASCVD events, indicating that the dose might not be critical (Haley and Chessman, 2020).

### Platelet Receptor Gene Polymorphism

Platelet receptor gene polymorphism is considered to be closely related to platelet activation, adhesion, aggregation, and the development of thrombotic diseases (Haybar et al., 2018). Singh, S. et al. explored the expression of MiR-19b-1-5p from the buffy coat of 945 patients with acute coronary syndrome (ACS) through reverse transcription quantitative polymerase chain reaction (PCR). Platelet function was detected by multiplate aggregometry testing. The results showed that after adjusting for age, gender, race, and history of previous stroke, platelet aggregation continues in the presence of aspirin [-Log-MiR-19b-1-5p (unstandardized beta, 44.50; 95% CI, 2.20–86.80; *p* < 0.05)], illustrating that AR is related to the lower expression of MiR-19b-1-5p (Singh et al., 2021). Wang, J. et al. evaluated the expression of long chain non-coding RNA H19 in ischemic stroke patients in order to explore the relationship between H19 and AR. 150 acute ischemic stroke patients were recruited, and urine 11-dehydrogenation thromboxane B2 (11dhTXB2) level, plasma H19, and 8-iso-prostaglandin F2α (8-iso-PGF2α) levels were determined. The results showed that plasma H19 levels were elevated significantly in patients with AR (*p* = 0.0203). The H19 level was positively related with urine 11dhTXB2/creatinine (R = 0.04364, *p* = 0.0106) and 8-iso-PGF2α (R = 0.04561, *p* = 0.0089). It was considered that H19 might induce AR by increasing the production of 8-iso-PGF2α (Wang et al., 2020). Xue, M. et al. explored genetic markers in Chinese patients with chronic stable angina pectoris (SAP) after percutaneous coronary intervention (PCI). 207 subjects were recruited to receive 100 mg aspirin daily. The inhibitory effect of platelets was evaluated by LTA. TXB2 and hypersensitive C-reactive protein (hs-CRP) were determined by radioimmunoassay. Genotyping was performed using Taqmanprobe technology (rs5787 and rs5911) and gene sequencing technology (rs3842788). The results showed that rs5911 A/C, C/C and A/A genotype (OR = 5.546, 95% CI = 1.812–11.404), and rs3842788 A/G and G/G genotype (OR = 8.358, 95% CI = 2.470–28.286) were correlated with AR. TXB2 and hs-CRP were significantly increased in the AR group, and the plasma TXB2 level was significantly increased in rs3842788a/G genotype carriers. This suggested that rs5911 and rs3842788 were specific genetic markers for AR in Chinese patients with chronic SAP (Xue et al., 2017).

### Increased Turnover of Platelet

The regeneration of platelet COX-1 improves the ability of circulating platelet pools to produce TXA2 and then promotes platelet aggregation (Floyd and Ferro, 2014). High platelet turnover can be triggered by reduction of abnormal megakaryocytopenia from primary thrombocythemia or peripheral consumption caused by inflammation (Coccheri, 2012). Restoration of thromboxane synthesis capacity in circulating blood reflects the number of platelets with uninhibited COX activity by aspirin. Data had shown that the time during which platelets with normal COX activity entered the circulation in diabetic patients was shorter than that of healthy individuals (Di Minno, 2011). Grove, E. L. et al. investigated the effect of platelet turnover on the antiplatelet effect of aspirin in 177 patients with stable CHD. Platelet turnover was measured by immature platelets and thrombopoietin. Platelet aggregation was measured using the VerifyNow aspirin test and MEA. The results showed that the level of immature platelets was closely related to MEA (*r* = 0.31–0.36, *p* < 0.0001), and sP-selector which is the marker of platelet activation (*r* = 0.19, *p* = 0.014). The antiplatelet effect of aspirin decreased within the increase of the platelet turnover rate, and AR was obvious consequently (Grove et al., 2011). The clinical association between accelerated platelet turnover and AR increased the risk of thrombotic disease events (Di Minno et al., 2012).

### Inflammation

Chronic inflammation is the underlying pathological mechanism of atherosclerosis-induced CVDs. The complex process involves the interaction of vascular endothelial cells, immune cells, and molecular transmitters, and promotes the development of atherosclerotic thrombotic disease by activating the rupture of atherosclerotic plaque (Shah, 2019). Inflammatory cytokines contribute to the formation of AR through platelet transformation, activation, and adhesion processes (Du et al., 2016). The pre-thrombotic state can be generated by the production and release of thromboxane A2 due to the increased expression of cyclooxygenase-2, which is associated with inflammation (Yalcinkaya and Celik, 2014). Reactivity to aspirin therapy may be reduced by inflammation-induced AR (Shan et al., 2010).

### Other Mechanisms

Kyyak, Y. H. et al. observed the functional status and ultrastructure of platelets in 36 patients with ACS under electron microscopy. The majority of platelets were found to be under an activated state, with pseudopedence, partial platelet aggregation, osmiophilism, vacuolation, and even calcification and apoptosis. Therefore, the researchers considered that AR may be caused by the degenerative changes and calcification of platelets (Kyyak et al., 2019).

Recent studies have shown that 8-isoprostaglandin F2α induced by oxidative stress can mediate the occurrence of AR. 8-Isoprostaglandin F2α as an agonist binding to the thromboxane platelet receptor and promoting vasoconstriction and platelet activation. 8-Isoprostaglandin F2α produced by the lipid peroxidation pathway is independent of COX activity, which means it is not affected by aspirin (Bauer et al., 2014). Therefore, platelet aggregation still occurs after TXA2 is inhibited by aspirin and leads to AR (Guo et al., 2019).

Tan, C. et al. observed platelet function and serum ubiquitin levels in 250 patients with AMI before and after taking aspirin to explore the possible mechanism of AR. They found that AR occurred in 47 patients, and serum ubiquitin levels were higher in AR patients than in healthy patients. Patients with high serum ubiquitin levels showed high levels of platelet ubiquitination, ubiquitinated proteins, and ubiquitinated cyclooxygenase-1. Serum ubiquitin promoted COX-1 ubiquitination through the CXC chemokine receptor 4 (CXCR4) pathway and prevented aspirin from acetylation of its target *in vitro* studies, thereby reducing the antiplatelet effect of aspirin, which might be involved in the mechanism of AR (Tan et al., 2015). In addition, there are also drug interactions and the influence of patient comorbidities in clinical practice which deserve more exploration.

### Current Clinical Researches on AR

Olas, B. investigated the antiplatelet effects of fish and vegetable oils and their constituent fatty acids. Studies in patients with a variety of CVDs have shown that both fish and vegetable oils contain protective components with antiplatelet activity. And, vegetable oils contain compounds known as phytosterols that protect the heart from hypercholesterolemia. Therefore, the author considered that vegetable oils might play a key role in the prevention and treatment of CVDs associated with platelet overactivation (Olas, 2020). Al-Azzam, S. I. et al. conducted a cross-sectional study of 418 patients taking aspirin and found that the use of statin was associated with the improvement of AR (Al-Azzam et al., 2012). Flavonoids have been found to have antiplatelet activity. In recent years, they have been focused on research and considered favorable drug candidates in the future (Khan et al., 2018). Some researchers considered that lifestyle changes such as smoking cessation, exercise, and weight loss might improve aspirin response, and prevention and treatment of complications related to AR such as hyperlipidemia, diabetes, and hypertension might also help improve AR (Kasmeridis et al., 2013; Ardeshna et al., 2019). In addition, other antiplatelet drugs, such as clopidogrel and P2Y12 inhibitor ticagrelor in combination with aspirin have also been studied (Divani et al., 2013). TCM treatment is based on a holistic perspective and treats the patient as a whole rather than just targeting a certain disease. In recent years, studies on the improvement of AR by TCM have been paid more and more attention, and the advantages of TCM have been reflected in clinical studies (Table 1) (Chen, 2019).

**TABLE 1:** Research studies on improving aspirin resistance by blood-activating herbs.

Intervention	Number of subjects	Findings	References
TCM for promoting blood circulation and removing blood stasis	1,055 subjects	Incidence of AR↓; the maximum platelet aggregation rate induced by ADP and AA↓	Guan et al. (2020)
Blood-activating TCM combined with aspirin	327 subjects	Platelet aggregation rate induced by ADP and AA↓; adverse events and endpoint events were low	Liu et al. (2016)
TCM extracts with water, 90% ethanol, and ethyl acetate	31 kinds of TCM	Chuanxiong (Rhizoma Chuanxiong), yanhusuo (*Rhizoma Corydalis yanhusuo*), and Danshen (*Radix Salvia miltiorrhiza*) had similar or higher antiplatelet aggregation effect than aspirin	Chen and Chou (2017)
Assess the efficacy and safety of Chinese herbal medicine for AR	1,011 subjects	Tongxinluo capsule and Danshen-based prescriptions were the most frequently used herbal prescriptions, while Danshen root, milkvetch root, leech, and rosewood were the most frequently used single herbs	Liu et al. (2014)
SMDS extract from *Salvia miltiorrhiza*–combined aspirin	135 patients with SAP	Improvement of AA% sensitivity of the SMDS-combined aspirin group is the highest; TCM symptoms of the SMDS-combined aspirin group are higher than those of the aspirin group	Lyn et al. (2021)
Compound Danshen Dripping Pills combined with aspirin	40 patients with CHD	Platelet aggregation rate induced by ADP and AA↓	Lin (2016)
Compound Danshen dripping pills (10 grains, tid) with aspirin (100 mg/d)	72 patients with SAP	Platelet aggregation rate↓	Chen (2019)
Danhong injection in combination with aspirin	100 CHD patients with AR	The rate of platelet aggregation, salicylic acid levels, the accumulation of salicylic acid, TXB2, TXB2/6-keto-PGF1α↓; plasma CAT, GPx, plasma SOD activity, and serum G-17 levels↑	Wang and Xu (2018)
Danhong injection combined with aspirin	50 CHD patients with AR	Platelet reactive units and readmission rate↓	Wang et al. (2017)
Tongxinluo capsule combined with aspirin	330 CHD patients	Platelet aggregation values induced by COL and ADP↓	Yin et al. (2010)
Naoxintong capsule combined with aspirin	151 elderly Chinese patients with NVAF and VKORC1 gene variation	Incidence of severe bleeding↓	Wang et al. (2016)

TCM, traditional Chinese medicine; ADP, adenosine diphosphate; AA, arachidonic acid; AR, aspirin resistance; SAP, stable angina pectoris; *Salvia miltiorrhiza* depside salt, SMDS; arachidonic acid induction rate, AA%; SAP, stable angina pectoris; TXB2, thromboxane B2; catalase, CAT; GPx, glutathione peroxidase; superoxide dismutase, SOD; gastrin-17, G-17; collagen, COL; NVAF, non-valvular atrial fibrillation; vitamin K epoxide reductase, VKORC.

## Researches on Improving AR by Blood-Activating Herbs Combined With Aspirin

Low compliance, insufficient dose, and the interaction between non-steroidal inflammatory drugs (NSAIDs) and platelets are important factors leading to AR and the risk of thrombus. Blood-activating herbs have good effects on improving microcirculation, antiplatelet aggregation, and anti-thrombosis in CVDs (Wang et al., 2014).

A systematic review of 1,055 subjects studying the improvement of AR by TCM for promoting blood circulation and removing blood stasis showed that the incidence of AR [RR = 0.41, 95% CI (0.32,0.52), *p* < 0.00001], the maximum platelet aggregation rate induced by ADP [MD = −6.20, 95% CI (−7.83, −4.57),*p* < 0.0001], and the maximum platelet aggregation rate induced by AA [MD = −4.8,95% CI (−8.16, −1.44), *p* = 0.005] were significantly decreased after treatment, indicating a very positive efficacy of blood-activating herbs in improving AR (Guan et al., 2020). Liu et al. conducted a meta-analysis of the RCTs of blood-activating TCM combined with aspirin versus aspirin alone in the treatment of AR and found that the combined therapy significantly reduced the ADP-induced platelet aggregation rate [SMD = −1.78, 95% CI (−2.95, 0.61), *p* < 0.003] and AA-induced platelet aggregation rate [SMD = −2.31, 95% CI (−3.41, −1.21), *p* < 0.0001], and there was no increase in endpoint events [R = 0.26, 95% CI (0.05, 1.35), *p* > 0.05], indicating the good safety of blood-activating TCM (Liu et al., 2016). Chen Cen. et al. prepared 31 kinds of TCM extracts with water, 90% ethanol, and ethyl acetate. The antiplatelet aggregation effects of various TCM for blood activation and stasis removal were measured on the platelet aggregator *in vitro* using 5′-ADP, bovine thrombin, and AA as inducers, respectively, and aspirin was taken as the positive control. It was found that *Chuanxiong* (*Rhizoma Chuanxiong*), *yanhusuo* (*Rhizoma Corydalis yanhusuo*), and *Danshen* (*Radix Salvia miltiorrhiza*) had at least similar or higher antiplatelet aggregation effect than the aspirin group (Cen et al., 2017). Liu et al. conducted a systematic review of 16 RCTs with a total of 1,011 subjects to evaluate the therapeutic effect of Chinese herbal medicine on AR. The results indicated that *Tongxinluo* capsules and prescriptions based on *Danshen* (*Radix Salvia miltiorrhiza*) were the most commonly used TCM prescriptions, and the most commonly used single TCM included *Danshen* (*Radix Salvia miltiorrhiza*), *Leech* (*Whitmania pigra Whitman*), and *Rosewood* (*Pterocarpus indicus Willd*). It was suggested that Chinese herbal medicines as potential agents for improving AR merit further more rigorous designs of RCTs to provide further evidence (Liu et al., 2014).

Lyu, J. et al. explored the curative effect of the Salvia Miltiorrhiza Depside Salt (SMDS) extract from *Danshen* (*Radix Salvia miltiorrhiza*) for SAP. A total of 135 subjects were randomly assigned to the SMDS group, aspirin group, and SMDS-combined aspirin group. Thromboelastography, visual analog scale score of TCM symptoms, and platelet aggregation detected by light transmittance aggregometry were determined at baseline and after 10-day treatment, respectively. The results showed that the improvement of arachidonic acid induction rate (AA%) sensitivity of the SMDS-combined aspirin group was the highest among the three groups [*p* < 0.001, 95% CI (0.00–0.00)], and TCM symptoms of the SMDS-combined aspirin group was higher than those of the aspirin group [MD = 1.71, 95% CI (0.15–3.27), *p* = 0.032]. Those findings indicated that SMDS combined with aspirin is an effective intervention for SAP (Lyu et al., 2021).

Research on compound *Danshen* dripping pills combined with aspirin in treating patients with CHD showed that the platelet aggregation rate induced by ADP and AA was lower than that of aspirin alone (74.2 ± 1.4 vs. 70.1 ± 1.3, *p* < 0.05; 26.4 ± 5.3 vs. 24.3 ± 3.1, *p* < 0.05), illustrating the high clinical application value (Lin, 2016). Chen et al. recruited 72 patients with SAP who took aspirin (100 mg/d) continuously for more than 4 weeks and were confirmed as AR by thrombelastography. The control group took 100 mg/d orally according to the original dose, and the experimental group took compound *Danshen* dripping pills (10 grains, tid) additionally. After 1-month treatment, the platelet aggregation rate of the experimental group was significantly lower than that of the control group [(69 ± 6)% vs. (44 ± 5)%, *p* < 0.05], suggesting that the compound *Danshen* dripping pill has a preferable effect on reducing platelet aggregation (Si-rui et al., 2016).

Wang et al. observed the effect of *Danhong* injection on the antiplatelet effect of aspirin and gastric mucosa damage in patients with CHD. *Danhong* injection in combination with aspirin decreased the rate of platelet aggregation, the salicylic acid levels, and the accumulation of salicylic acid, TXB2, and TXB2/6-keto-PGF1α. The activity of plasma catalase (CAT), glutathione peroxidase (GPx), and plasma superoxide dismutase (SOD), and the serum gastrin-17 (G-17) level were higher than those of the control group (*p* < 0.05), suggesting that the combination of aspirin strengthened the inhibition of COX-1, promoted gastric mucus secretion, and enhanced the body’s antioxidant capacity (Wang and Xu, 2018). In addition, *Danhong* injection combined with aspirin was also found to reduce platelet reactive units (540.39 ± 54.39 vs. 654.49 ± 39.48, *p* < 0.01.) and readmission rate (20 vs. 40%, *p* = 0.029) in CHD patients with AR (Wang et al., 2017).

Yin et al. observed the *Tongxinluo* capsule on platelet aggregation in AR patients with CHD. Platelet aggregation values were determined by collagen (COL) and ADP as inducers. Results showed that after 1-month treatment, platelet aggregation values of the *Tongxinluo* capsule group and *Tongxinluo* capsule–combined aspirin group were significantly decreased, while those of the aspirin group did not, suggesting that *Tongxinluo* capsule inhibits the function of platelets and prevented the progress of the disease (Yin et al., 2010).

Wang et al. compared the effect of *Naoxintong* capsule combined with aspirin with warfarin at adjusted doses on thrombus risk in elderly Chinese patients with non-valvular atrial fibrillation (NVAF) and vitamin K epoxide reductase (VKORC1) gene variation. It was observed that combination therapy reduced the incidence of severe bleeding (0 vs. 7.9%, OR: 0.921, 95% CI: 0.862–0.984, *p* = 0.028), demonstrating the antithrombotic effect of blood-activating herbs (Wang et al., 2016).

## Potential Mechanisms on Improving AR by Blood-Activating Herbs Combined With Aspirin

The mechanism of traditional Chinese medicine to improve aspirin resistance is not clear. The studies of Chinese herbal medicine that have been tested for aspirin resistance are shown in Table 2.

**TABLE 2:** Research studies on Chinese herbs that have been tested for aspirin resistance.

Chinese herb	Scientific name	Chemical composition	Findings	References
*Chuanxiong*	*Rhizoma Chuanxiong*	Chuanxiongzine	ADP-, AA-, and THR-induced platelet aggregation↓	Cen et al. (2017)
*Huaihua*	*Flos Sophorae*	Rutin, sophorae glycol	Stronger anti-aggregation effect even than that of aspirin	
*Niuxi*	*Radix Achyranthis Bidentatae*	Polysaccharide, saponin, and sterone	Strong anti-aggregation effect at lower concentrations with THR and AA as aggregation inducers	
*Chishao*	*Radix Paeoniae Rubra*	Paeoniflorins and paeonols	Strong anti-aggregation effect at lower concentrations with AA as the aggregation inducer	
*Danshen*	*Radix Salvia miltiorrhiza*	Salvia miltiorrhiza depside salt	Rate of sensitivity in AA%↑	Lyu et al. (2021)
*Danhong* injection	*Radix Salvia miltiorrhiza* and *Carthamus tinctorius L*	Tanshinone, phenolic acid, safflor yellow pigment, and flavone	Platelet aggregation rate, TXB2 and TXB2/6-keto-PGF1α↓	Wang and Xu (2018)
*Danhong* injection	*Radix Salvia miltiorrhiza* and *Carthamus tinctorius L*	Tanshinone, phenolic acid, safflor yellow pigment, and flavone	Platelet reactive units and readmission rate↓	Wang et al. (2017)
Compound *Danshen* Dropping Pills	*Radix Salvia miltiorrhiza*	Water-soluble Danshen	AA-induced platelet aggregation rate↓	Zhang (2015)
Tongqiao Huoxue Decoction	—	—	ADP- and AA-induced platelet aggregation rate↓	Liu (2019)
*Ginkgo biloba*	*Ginkgo biloba L*	Ginkgo flavone and ginkgolide	ADP- and AA-induced platelet aggregation rate↓	Feng et al. (2016)
*Tongxinluo* capsule	—	—	ADP- and AA-induced platelet aggregation↓	Yin et al. (2010)
*Naoxintong* capsule	—	—	Incidence of severe bleeding↓	Wang et al. (2016)
*Xuefu Zhuyu* Capsule	—	Peach kernel water extract, safflor yellow pigment, tangerine peel, and saikosaponin a	Platelet aggregation rate↓	Zhu et al. (2018)
*Taoren-Honghua*	*Persicae Semen* and *Carthamus tinctorius L*	Amygdalin and hydroxysafflor yellow A	WBV, PV and PCV↓, TT, APTT and PT↑, FIB↓	Liu et al. (2012)

AA, arachidonic acid; ADP, adenosine diphosphate; THR, thrombin; WBV, whole blood viscosity; PV, plasma viscosity; PCV, packed cell volume; TT, prolonged thrombin time; APTT, activated partial thromboplastin time; PT, prothrombin time; FIB, fibrinogen content.

Lai et al. explored the potential active ingredients and mechanism of *Danhong* injection in improving AR based on network pharmacology. The Traditional Chinese Medicine Database and Analysis Platform was used to collect the main active components and action targets of *Danshen* (*Salviae miltiorrhizae radix et rhizome*) and *Honghua* (*Carthamus tinctorius L.*). The main active ingredients were screened, and 60 active ingredients were obtained, including 51 components of *Danshen* (*Salviae miltiorrhizae radix et rhizome*), 11 components of *Honghua* (*Carthamus tinctorius L.*), two components of *Danshen* (*Salviae miltiorrhizae radix et rhizome*) and *Honghua* (*Carthamus tinctorius L.*), and 159 target genes. The results of gene enrichment analysis suggested that *Danhong* injection mainly improved AR through multicomponent, multi-target, and multichannel pathways involving coagulation process, inflammatory response, and metabolic regulation (Lai et al., 2019).

Xue et al. observed the effect of *Xuefu Zhuyu* Capsule on AR and explored its mechanism. Patients with AR were randomly divided into three groups: aspirin high-dose group (300 mg/d, qd), *Xuefu Zhuyu* Capsule group (six grains each time, bid), and *Xuefu Zhuyu* Capsule (six grains each time, bid) in combination with aspirin (100 mg/d, qd). AA and platelet aggregation rate, TXB2, 6-Keto-prostaglandin F 1 alpha (6-Keto-PGF_1α)_, and hs-CRP induced by ADP were measured. The platelet aggregation rate in the combination group (AA-induced: 23.57 ± 4.1 vs. 25.76 ± 3.76; ADP-induced: 72.18 ± 7.57 vs. 77.01 ± 9.83), TXB2 (279.81 ± 52.49 vs. 304.53 ± 47.3), and Hs-CRP (3.69 ± 0.99 vs. 4.3 ± 1.24) was significantly lower than that in the aspirin group. The effect of increasing 6-Keto-PGF_1α_ in the combination group was better than that of the high-dose aspirin group. The mechanism of *Xuefu Zhuyu* Capsule to improve AR was considered to be associated with TXB_2_, hs-CRP, and 6-Keto-PGF_1α_ (Zhu et al., 2018).

*Taoren* (*Persicae Semen*) and *Honghua* (*Carthamus tinctorius L.*), which is also called *Taoren-Honghua* (TH), is a commonly used herb group for promoting blood circulation and removing blood stasis in TCM. Liu et al. explored the effects of its main components amygdalin and hydroxysafflor yellow A (HSYA) on hemorheology in rats. The intervention methods include TH, amygdalin, HSYA, amygdalin combined with HSYA, and aspirin. Rats were administered every 12 h. After the fifth administration, during the interval between the two injections of adrenaline hydrochloride, the rats except the control group with blood stasis syndrome were placed in ice water to establish the model; blood samples were collected 30 min after the last administration the next day. The results indicated that TH significantly reduced whole blood viscosity (WBV), plasma viscosity (PV), and packed cell volume (PCV); prolonged thrombin time (TT), activated partial thromboplastin time (APTT), and increased prothrombin time (PT); and decreased fibrinogen content (FIB). Amygdalin mainly reduced PV and FIB and prolonged APTT while HSYA mainly reduced WBV and PV. This reveals that TH plays a synergistic role in reducing blood viscosity and improving coagulation parameters (Liu et al., 2012).

## Conclusion and Prospective

Platelet activation and coagulation regulation play an important role in vascular injury and atherosclerotic thrombus events (Tomaiuolo et al., 2017). Aspirin as an antiplatelet drug is an important part of the secondary prevention of atherosclerotic thrombosis; however, the antiplatelet aggregation effect of aspirin is different among the crowd. AR is defined as the state in which platelet reactivity does not reach the ideal reduction after aspirin treatment (Ping-ping et al., 2019). Research had shown that AR is an independent predictor of cardiovascular adverse risk (Pasala et al., 2016). In recent years, more and more studies have been conducted on AR, and the improvement of AR may become a research hot spot in the future (Xue et al., 2014; Al-Jabi, 2017; AJin et al., 2019; Baş et al., 2020).

It has been found that the combination of blood-activating herbs and aspirin can improve AR and reduce the rate of platelet aggregation (Youlong and Zhongjun, 2014). Monomers including *Danshen* (*Radix Salvia miltiorrhiza*) and *Honghua* (*Carthamus tinctorius L.*) and compounds including *Danshen* Dropping Pills, *Danhong* injection, *Tongxinluo* capsule, *Naoxintong* capsule, and *Xuefu Zhuyu* capsule have advantages in antiplatelet aggregation, which were the research hot spots in recent years. However, the effectiveness of blood-activating TCM in CVDs still needs further rigorous large-scale clinical trials to provide further strong evidence. There are many studies on the role of blood-activating traditional Chinese medicine, but scattered studies have not formed a unified therapeutic target network. Researchers have used different computational approaches to construct network models of Chinese herbal medicine to explore the interaction of ingredients of disease pathways (Jafari et al., 2020; Wang et al., 2021). The network model construction of Chinese herbal medicine may further reveal the intervention targets and potential treatment orientation of diseases. The mechanism of blood-activating herbs on improving AR remains unclear, and there is still a lack of relevant research at home and abroad. Studies have found that blood-activating herbs may improve AR through multicomponent, multi-target, and multichannel pathways such as coagulation process, inflammatory reaction, and metabolic regulation, which are worthy of further exploration and may become a potential target for future treatment.
